# Long-term endovascular treatment of the thoracic aorta: an observational study

**DOI:** 10.1590/1677-5449.202500312

**Published:** 2025-06-02

**Authors:** Silvio Rogério Moura de Araújo, Edenilson de Souza Teixeira, Mario Giovanne Fernandes Duarte, Luana Vieira de Medeiros Santana, Hideki Zimermann Kamitani, Vanessa Ellen Silva Carmo, Pedro Pereira Tenório

**Affiliations:** 1 Universidade Federal do Vale do São Francisco, Paulo Afonso, BA.

Dear Editor,

We congratulate Brandi et al.^1^ for their article “Long-term outcomes after endovascular aortic treatment in patients with thoracic aortic diseases”. The study analyzed the long-term results of endovascular treatment in patients with diseases of the thoracic aorta treated with self-expanding stent-grafts. However, we identified certain points that merit discussion.

The heterogeneity of the patients’ conditions makes generalization of results and identification of specific patterns in each subset difficult. The high proportion of patients with type B dissection (83.3%) skews the results in the direction of this condition, while less frequent diseases receive less attention. Studies of type B dissection demonstrate that thoracic endovascular aortic repair (TEVAR) improves survival and reduces mortality compared with clinical treatment alone over a 5-year horizon,^2^ corroborating the findings of this study.^1^ However, the absence of stratification by disease could cause confusion and yield imprecise clinical data.

The lack of a control group limits comparison of the efficacy and safety of endovascular treatment with other approaches. Despite its possible advantages, such as reduced morbidity and early mortality, medium- and long-term outcomes for diseases of the thoracic aorta remain inconclusive.^3^ Moreover, treatment success is dependent on an effective initial procedure and patient adherence to regular follow-up,^4^ which raises doubts about the long-term benefits, considering the rigorous follow-up needed. Inclusion of comparison groups or use of statistical methods could reduce the selection bias and improve the analysis.

Chronic renal failure was identified as a risk factor for mortality. However, a more robust multivariate analysis could reveal interactions between clinical factors and outcomes, especially with regard to systemic arterial hypertension (SAH) and diabetes mellitus (DM). For example, type 2 DM has been associated with reduced mortality and fewer complications after TEVAR for type B aortic dissection.^5^ In turn, SAH can contribute to both aortic and cardiac damage.^6^

Finally, it is very important to extend the analysis of the impact of the heterogeneous nature of the sample and of technological developments on the results. Use of older endovascular devices could have contributed to a higher incidence of complications.
