# Supplementary material for: Long-term endovascular treatment of the thoracic aorta: an observational study
Source: J Vasc Bras. 2025 Jun 2;24:e20250031. doi: 10.1590/1677-5449.202500312 (PMC12166754; doi:10.1590/1677-5449.202500312)
Supplement: Translation

# Translation: Tratamento endovascular da aorta torácica a longo prazo: estudo observacional

**DOI:** 10.1590/1677-5449.202500311

**Authors:** Silvio Rogério Moura de Araújo, Edenilson de Souza Teixeira, Mario Giovanne Fernandes Duarte, Luana Vieira de Medeiros Santana, Hideki Zimermann Kamitani, Vanessa Ellen Silva Carmo, Pedro Pereira Tenório

**Affiliations:** 1 Universidade Federal do Vale do São Francisco, Paulo Afonso, BA, Brasil.

Prezado Editor,

Congratulamos Brandi et al.^1^ pelo artigo “Long-term outcomes after endovascular aortic treatment in patients with thoracic aortic diseases”. O estudo analisou os resultados a longo prazo do tratamento endovascular em pacientes com doenças da aorta torácica tratados com *stent-grafts* autoexpansíveis. Entretanto, identificamos alguns pontos que merecem discussão.

A heterogeneidade das condições dos pacientes dificulta a generalização dos resultados e a identificação de padrões específicos para cada subgrupo. A alta proporção de pacientes com dissecção do tipo B (83,3%) direciona os resultados para essa condição, enquanto doenças menos frequentes recebem menor destaque. Estudos sobre a dissecção do tipo B demonstram que o reparo aórtico endovascular torácico (TEVAR) melhora a sobrevida e reduz a mortalidade em comparação ao tratamento clínico isolado em 5 anos^2^ , corroborando os achados desse estudo^1^ . Contudo, a ausência de estratificação por doença pode gerar confusão e fornecer dados clínicos imprecisos.

A ausência de um grupo-controle limita a comparação da eficácia e segurança do tratamento endovascular com outras abordagens. Apesar das possíveis vantagens, como menor morbimortalidade precoce, os desfechos em médio e longo prazos para doenças da aorta torácica seguem inconclusivos^3^ . Ademais, o sucesso do tratamento depende de um procedimento inicial eficaz e da adesão do paciente a seguimentos regulares^4^ , o que levanta dúvidas sobre os benefícios a longo prazo, considerando o rigor exigido no acompanhamento. A inclusão de grupos comparadores ou o uso de métodos estatísticos poderia reduzir o viés de seleção e aprimorar a análise.

A insuficiência renal crônica foi identificada como fator de risco para mortalidade. Todavia, uma análise multivariada mais robusta poderia revelar interações entre os fatores clínicos e os desfechos, especialmente em relação à hipertensão arterial sistêmica (HAS) e ao diabetes melito (DM). O DM tipo 2, por exemplo, tem sido associado a menor mortalidade e a menos complicações na dissecção aórtica tipo B após o TEVAR^5^ . Já a HAS pode contribuir para danos aórticos e cardíacos^6^ .

Por fim, é fundamental aprofundar a análise do impacto da heterogeneidade da amostra e da evolução tecnológica nos resultados. O uso de dispositivos endovasculares antigos pode ter contribuído para a maior incidência de complicações.
