# Supplementary material for: Long-term endovascular treatment of the thoracic aorta: an observational study
Source: J Vasc Bras. 2025 Jun 2;24:e20250031. doi: 10.1590/1677-5449.202500312 (PMC12166754; doi:10.1590/1677-5449.202500312)
Supplement: Related texts

# Reply: RESPONSE LETTER

**Authors:** Antonio Carlos Brandi, Carlos Alberto dos Santos, Josélia Menin Brandi, Marcio Antonio dos Santos, Paulo Henrique Husseine Botelho

**Affiliations:** 1 Faculdade de Medicina de São José do Rio Preto FAMERP, São José do Rio Preto, SP, Brasil.; 2 Faculdade de Medicina de Marília FAMEMA, Marília, SP, Brasil.

Dear Editor,

All of the questions and doubts raised with respect to this article are pertinent and objective. In fact, some of them were discussed when this work was presented to a cardiology and cardiovascular surgery conference.

As an initial explanation of this study, we employed an observational, prospective study design, conducted in accordance with the clinical study guidelines set out in the STROBE checklist, as described in the study methodology.

With relation to the heterogeneous nature of the aortic diseases included in the study, we did consider the possibility of only using data from patients with aortic dissections, which would have resulted in a “cleaner” article, reducing the number of variables in the clinical results. However, since our basis for comparison was the results of conventional surgery to treat diseases of the descending thoracic aorta (references 4, 12, 15, 17, and 19) and the surgical procedure adopted for these diseases is basically the same (the elephant trunk), we decided to include all of the patients.

The variability of aortic diseases was also mentioned in the article limitations: “*Nevertheless, it analyzes a dataset from a large series of endovascular treatments in patients with thoracic aortic diseases and it was appropriate to include these patients together to make it possible to extract results and conclusions from this* ***heterogeneous patient population*** . *In addition, the results reflect outcomes from a real institutional setting, showing the evolution of cases over 132 months of follow-up*”.

As mentioned above, when comparing data, in lieu of controls, we used published data on open surgery to treat the descending aorta. Primary success of endoprosthesis implantation and also the rates of complications and hospital mortality were also compared with specific articles. The best results were observed up to 60 months of follow-up.

We strive to ensure rigorous outpatients follow-up in both the immediate and late postoperative periods, with control tomography scans scheduled in advance, in line with a pre-established protocol. Some of the return consultations and imaging examinations were missed, despite proactively seeking patients via telephone contact. However, the major problem linked to later complications was failure to control systemic arterial pressure.

Among risk factors for mortality, we did not distinguish between diabetes types 1 and 2. However, chronic renal failure, and its harmful effects on the entire cardiovascular system, was the number one factor associated with postoperative mortality.

There is no doubt that the progressive development of endoprostheses contributed significantly to improving the results. When we started using surgery to correct diseases of the thoracic descending aorta (thoracic aortic endovascular repair, TEVAR) in 1998, the only endoprostheses we had available were stainless steel devices made by Braile-Biomédica. Over the years, both manufacturers and medical teams followed a learning curve. In 2004-2005, nitinol endoprostheses were launched by Braile-Biomédica, which yielded technical improvements and better clinical results.

In the study discussed here, 7 of the 9 patients who had type I endoleaks over the course of medical follow-up had been fitted with stainless steel devices. In 2013, our hospital authorized the use of imported endoprostheses, enabling us to treat more complex lesions, with improved comfort and better results. Now, we are close to reaching a total of 600 patients who have undergone endovascular surgery of the thoracic aorta.

# Reply: CARTA RESPOSTA

**Authors:** Antonio Carlos Brandi, Carlos Alberto dos Santos, Josélia Menin Brandi, Marcio Antonio dos Santos, Paulo Henrique Husseine Botelho

**Affiliations:** 1 Faculdade de Medicina de São José do Rio Preto FAMERP, São José do Rio Preto, SP, Brasil.; 2 Faculdade de Medicina de Marília FAMEMA, Marília, SP, Brasil.

Caro Editor,

Todos os questionamentos e dúvidas levantados sobre este artigo são pertinentes e objetivos. Alguns deles, inclusive, já foram discutidos durante a apresentação deste trabalho em congressos de cardiologia e cirurgia cardiovascular.

Como explanação inicial deste trabalho, adotamos um modelo de estudo observacional prospectivo, conduzido de acordo com as diretrizes de pesquisa clínica estabelecidas pelo STROBE, conforme descrito na metodologia.

Com relação à heterogeneidade das doenças da aorta incluídas no estudo, também consideramos a possibilidade de utilizar apenas os dados dos pacientes com dissecção da aorta, o que deixaria o artigo mais “limpo” e reduziria as variáveis nos resultados clínicos. No entanto, como tínhamos como comparativo os resultados das cirurgias convencionais para o tratamento das doenças da aorta torácica descendente (referências 4, 12, 15, 17 e 19) e o procedimento cirúrgico adotado nessas doenças era basicamente o mesmo (técnica da tromba de elefante), optamos por incluir todos os pacientes.

Essa variedade de doenças da aorta também foi citada nas limitações do artigo: “*Nevertheless, it analyzes a dataset from a large series of endovascular treatments in patients with thoracic aortic diseases and it was appropriate to include these patients together to make it possible to extract results and conclusions from this* ***heterogeneous patient population*** . *In addition, the results reflect outcomes from a real institutional setting, showing the evolution of cases over 132 months of follow-up*”.

Conforme mencionado anteriormente, utilizamos como controle, na comparação dos resultados, dados da literatura sobre cirurgias abertas na aorta descendente. O sucesso primário do implante da endoprótese, bem como as complicações e a mortalidade hospitalar, também foram comparados com artigos específicos. Os melhores resultados foram observados até 60 meses de seguimento.

Buscamos garantir um acompanhamento ambulatorial rigoroso nos períodos pós-operatório imediato e tardio, com tomografias de controle previamente agendadas, conforme protocolo estabelecido. Houve falhas em alguns retornos e na realização de exames, mesmo com a busca ativa por telefone. No entanto, o principal problema enfrentado em relação às complicações tardias foi a falta de controle da pressão arterial sistêmica.

Entre os fatores de risco para mortalidade, não realizamos a distinção entre os tipos 1 e 2 do diabetes. No entanto, a insuficiência renal crônica, com seu efeito deletério sobre todo o sistema cardiovascular, foi o principal fator associado à mortalidade pós-operatória.

Não há dúvidas de que a evolução das endopróteses contribuiu significativamente para a melhoria dos resultados. Quando iniciamos as cirurgias endovasculares para correção das doenças da aorta torácica descendente (reparo aórtico endovascular torácico, TEVAR) em 1998, dispúnhamos apenas das endopróteses de aço inox da Braile-Biomédica. Ao longo do tempo, houve uma curva de aprendizado tanto das indústrias quanto das equipes médicas. Em 2004-2005, foram lançadas as endopróteses de nitinol da Braile-Biomédica, o que resultou em uma melhora técnica e nos resultados clínicos.

Neste trabalho, dos 9 pacientes que apresentaram *endoleak* tipo I ao longo de todo o seguimento médico, 7 haviam recebido dispositivos de aço inox. Em 2013, o hospital autorizou o uso de endopróteses importadas, permitindo a abordagem de lesões mais complexas, com maior conforto e melhores resultados. Atualmente, contamos com quase 600 pacientes submetidos à cirurgia endovascular da aorta torácica.
